# Learning in Tension: A Case Study Examining What Internal Medicine Residents Learn in the Ambulatory Care Setting

**DOI:** 10.5334/pme.443

**Published:** 2023-01-20

**Authors:** David C. Thomas, Janneke M. Frambach, Pim W. Teunissen, Tamara Goldberg, Frank W. J. M. Smeenk

**Affiliations:** 1 Icahn School of Medicine at Mount Sinai, New York, NY, US; 2 Department of Medicine, Department of Medical Education and Department of Rehabilitation and Human Performance, US; 3 School of Health Professions Education (SHE), Maastricht, University, Maastricht, NL; 4 Department of Educational Development and Research, NL; 5 Department of Obstetrics & Gynecology, Maastricht University Medical Center, Maastricht, NL; 6 Department of Medicine and Department of Medical Education, US

## Abstract

**Introduction:**

Medical care of patients with complex conditions has shifted to the ambulatory setting, whereas current knowledge of resident learning is primarily based on studies from inpatient settings. Preparing trainees to adapt to this shift necessitates an understanding of what internal medicine (IM) residents currently learn during ambulatory rotations. The aim of this study is to identify what residents learn during their ambulatory care experience.

**Methods:**

Using a qualitative instrumental case study design, the authors conducted separate focus groups with IM trainees (n = 15), supervisors (n = 16), and program directors (n = 5) from two IM programs in New York City, USA in 2019. Participants were invited via email, and focus group sessions were complemented by document analysis of ambulatory syllabi.

**Results:**

Based on focus group commentary and document analysis, content learned in the ambulatory setting encompassed three domains; 1) patient needs, 2) the resident’s role within a healthcare team, and 3) health system opportunities and limitations. Residents also learned about tensions within and between these domains including the skills needed to care for patients versus the skills acquired, a desire for ownership of patient care versus fragmented care, and time allotted versus time required.

**Discussion:**

This study revealed two outcomes about what residents learn during their ambulatory care experience. First, learning content largely fell into three domains. Second, residents learned about the tensions between ideal care delivery and the realities of practice. These results highlight the imperative to better align curricula with clinical environments to meet the learning needs of residents.

## Introduction

The care of patients with complex illness has increasingly moved from the inpatient to outpatient setting, partly resulting from changing global demographics and innovations in medical technology [1]. Over the next half century, older patients in almost all countries will outnumber younger patients leading to increased visits [2]. In the United States, for instance, between 1999 and 2009 there was an increase in ambulatory care visits of almost 300 million per year or 3040 to 3720 visits per 1000 persons annually [3]. This trend results in more complex patients receiving care in the outpatient setting by internal medicine (IM) clinicians and trainees. Optimally preparing trainees to provide care for patients in this practice environment necessitates a curriculum that addresses both the medical and social complexities contributing to illness.

Despite growing recognition of the importance of ambulatory care training, there is limited description of what IM residents *actually learn* in ambulatory care [4]. Instead the literature describes what IM residents *should learn* in generalities and lacks granularity [4,5,6,7,8], and focuses on curriculum development and clinic redesign themes [9,10,11,12,13]. Although well recognized that learning in residency occurs in both inpatient and outpatient settings, research is lacking on what learning opportunities are available in ambulatory care and possibly different practice redesigns that reflect patients’ medical and social needs.

In IM residency, trainees are formally educated on a plethora of topics, including ambulatory care. This formal education is complemented by the informal curriculum described in the literature as unplanned and opportunistic [14]. Much of what residents learn is influenced by this informal curriculum as well as the hidden curriculum transmitted by behaviors, structures and implicit messages, operating at the level of organizational structure and culture [15]. What then are residents actually learning in ambulatory care with various curricula operating simultaneously? A socio-cultural perspective that considers the culture of the learning environment, the social nature of learning (i.e. learning occurring through interactions within and among the community), and learning in environments representative of real world practices [16] may help address this question. Research on residents’ learning in the inpatient setting has taken similar perspectives, and demonstrated the critical role of workplace-based learning for residents [17,18,19]and the importance of dialogue during practice [20]. Similarly, IM residents’ learning in ambulatory care occurs while caring for patients in a complex social process within the clinical learning environment [21]. Acknowledging the socio-cultural nature of learning can help identify what residents learn in the ambulatory setting, resulting from interactions with and between the formal, informal and hidden curricula.

With an increased focus on care delivery in the ambulatory setting, it is essential to know what and how residents in ambulatory care learn to enable setting up a training plan that is more informed and tailored to the context of the learning environment. As part of a series of studies to examine these issues [13,22] the aim of this case study is to understand *what* IM residents learn in the ambulatory care setting.

## Methods

### Design

We conducted an instrumental case study, which uses a real-life case to gain insight into a particular phenomenon [23,24], situated in a constructivist paradigm. This fits well with socio-cultural perspectives that focus on interactions between learners and their learning environment, and the active role of learners (and researchers) in creating knowledge. Using focus groups and document analysis, we investigated *what* IM residents learn in the ambulatory care setting (the phenomenon) using two IM residency programs (cases). The Icahn School of Medicine at Mount Sinai’s Institutional Review Board determined this study was “exempt human research” (HS#: 19-00475; GCO#: 19-0947(0001) ISMMS).

### Setting

Two IM residency programs at a large academic health system in New York City (NYC) were sampled purposively because of their strong emphasis on developing an advanced ambulatory care network, allowing rich data collection. The two sites were furthermore selected because they vary on a number of aspects: one is based at a medical school with a strong research portfolio across many domains (this program has 131 residents rotate in one ambulatory site) and the other is a freestanding hospital (this program has two ambulatory sites with 58 and 24 residents, respectively). Each program is based at a different urban teaching hospital and both are sponsored by the same medical school. Inpatient rotations run for six weeks followed by two weeks dedicated solely to the ambulatory setting. Residents return to the same outpatient clinic and inter-professional team to care for their own panel of patients.

### Participants

To gain a comprehensive picture of what residents learn in ambulatory care, three key participant groups, who likely offer complementary perspectives, were invited to participate by DCT, the study principle investigator, who serves as the Vice Chair for Education for the Department: 1) residents rotating on their ambulatory care block, 2) program directors and associate program directors, and 3) faculty who only supervise in ambulatory care. All members of the participant groups were invited via email to voluntarily attend role-specific focus groups. No incentives were offered and all who responded participated.

### Data collection and analysis

Focus groups were used to capitalize on participants’ interactions with each other. Residents (n = 15) and faculty (n = 16) had site-specific focus group meetings while the program directors (n = 2) and associate program directors (n = 3) from both sites met jointly in one group due to smaller numbers. TG, a faculty member on the research team with experience as an ambulatory educator who does not oversee the resident participants conducted the resident focus groups. TG and DCT conducted the other focus groups. All focus groups were conducted between June and September 2019. Each program has dedicated time for ambulatory seminars; focus groups were conducted during these, and not during patient care sessions. The focus groups were conducted using moderator guides (see Supplemental Content 1 (Moderator Guide for Resident Focus Groups) and Supplemental Content 2 (Moderator Guide for Faculty and Program Directors)). The sociocultural perspective served as a framework to develop the guides using both the literature and authors’ knowledge of ambulatory care. Focus groups were audio-recorded and transcribed verbatim, during which they were de-identified. Focus group data were analyzed using an iterative process and a constant comparative analysis approach to identify themes. The sociocultural framework served as a lens to identify themes. Two members of the research team (TG, DCT) independently reviewed the transcriptions. They discussed their own interpretations and developed a coding scheme that was applied to the whole data set. They categorized the codes, which served as the basis to develop themes, which were discussed and created with the team as a whole. We did not collect data until saturation, but we did feel the data were sufficiently rich to start data analysis and construct our results (theoretical sufficiency). We noticed overlap in the main topics between the focus groups with different groups of participants, suggesting that we captured the prominent and salient issues. Throughout the process, we looked for disconfirming evidence of the thematic structure we were developing.

To obtain a more robust view of the totality of what the residents’ learning looked like in the two cases, triangulation with the ambulatory care syllabi of the residency programs underwent content analysis [25,26]. Syllabi were chosen for this analysis as they list all topics to be covered during the three-year residency and represent the blueprint for the whole curriculum. For content analysis we categorized lectures into overarching themes for the first-year residents and separately for the second- and third-year residents. Ultimately, we looked at how the findings from the content analysis, as complimentary data, related to the themes identified.

### Statement of reflexivity

Reflexivity involves reflecting on a range of interactions between the researcher, project, including the participants, and how this affects the researchers’ choices and interpretations [27,28,29]. The main clinical and scholarly focus of DCT is in ambulatory care. DCT oversees all educational programs at both sites included in this case study and did not conduct any interviews with residents due to this supervisory role. JF is a medical education researcher with an expertise in qualitative research. TG is a director of a residency program and supervisor in ambulatory care. PT and FS are both physicians and medical education researchers having expertise in workplace-based learning. These backgrounds have shaped the study and research question. During the project, authors constantly asked each other why decisions were made and on what basis especially as our experiences influenced those decisions.

## Results

Through their ambulatory care rotations, we found that residents learned specific knowledge, skills and attitudes that fell into three domains: 1) patient needs, 2) their role within the healthcare team and 3) healthcare system opportunities and limitations. Additionally, we identified three areas of tension operating within and between these domains, which contributed to what they learned. These included the skills needed versus skills acquired to care for patients, a desire for ownership of patient care versus disjointed care, and the time allotted versus time required to care for patients in ambulatory care. In short, they learned that caring for their patients necessitates navigating their perceptions of ideal care delivery with the realities of practice. Below we elaborate on what residents learned about these domains and how the three tensions played out in the domains.

### Patient needs

The ambulatory setting provided an arena in which residents learn in real-time about the impact of social determinants of health on clinical care delivery and patient management decisions. Residents described that patients “*bring a lot of like not strictly medical issues to the visits and we’re not trained to really help or support them with those things and sometimes the visits are dominated by those issues and I don’t feel like I’m… well trained to help*” (FG-R2), indicating a tension between residents’ perception of the ideal medical encounter and reality of practice as it relates to patients’ needs. One faculty member noted, residents are:

“*learning about a lot of the non-medical, so to speak, things that affect health. So be it poverty or lack of access to care or lack of medical education or other education or complex family social issues and depression from broken families or from incarceration or from various things, substance abuse. They’re learning that all of these things are often as much or more the drivers of patient complaints than the classical things they learned about in medical school*” (FG-F2).

Residents discussed that supervisors are vital in teaching how to address social needs and cope with these issues as healthcare providers, suggesting that point-of-care learning often depends on the resident-supervisor relationship to assist with learning practical patient care skills.

This lack of preparedness to address the interwoven medical and social needs of patients underscores residents’ perception that the formal curriculum was often misaligned with the reality of their point-of-care clinical practice, both in terms of structure and content. This tension was recognizable in basic tasks, as one resident described needing “*someone [to] walk us through the steps that are needed to take to get someone new medical supplies and get them home health aide hours*” since these are “*really practical things that people need from us as their doctors that we have no idea about*” (FG-R2). From the content analysis of the syllabi, the vast majority of curriculum components, across all years of training, focused on ambulatory care medical knowledge. Nuances of medicine relating to specific patient populations came into play only during the upper-level years, as did professional development. What was a large focus of discussion during the focus groups, namely the social determinants of health, received little attention during the formal curriculum.

Additionally, within the patient needs domain, residents learned about challenges with shared decision-making, often perceiving a mismatch between patients’ concerns and their own agenda during the encounter. In describing a patient’s concern, a resident noted that “*it’s so not aligned with my priorities for the visit that I like can’t really focus on what they’re saying because I’m thinking like well, now I’m not going to get to talk to you about your glucose log and that’s very frustrating”(FG-R2)*. Time constraints revealed themselves while residents disentangled the components of the patient’s medical and social situations during a visit as they worked through the needed follow-up. The residents perceived a tension between the time required to see patients and allotted time, stating “*they’re very complex medically also and just is like impossible to do within the amount of time for some of those patients*” (FG-R2) so “you pick two things out of the ten…” (FG-R1). Through all of this, residents learned about the lives of their patients in a more comprehensive way.

### Role within the ambulatory healthcare team

The challenges of addressing patients’ complex needs in a set timeframe provided a path for residents to learn about their role within a larger healthcare team. Our data suggest residents learned how the roles and responsibilities of other team members is essential to both efficiency and quality of care. One resident observed about the clinic staff, “[*t]hey seem to understand the inner workings of the clinic…better than we do*” and teach us “what to do… to help facilitate their [the patient’s] care in the most efficient way possible” (FG-R1). They learned that team members “*really helped expedite the visits and they, like, sort of look out for things with the patient*” (FG-R2).

A tension arising within this domain was the desire of residents to assume ownership and accountability for patients while also recognizing the limitations of their own skillset and time. A program director noted, since “*a lot of our staff are there even when the residents are not*”, residents come to understand team members as essential threads of continuity who “*can update the resident on what’s happened sort of in the interim, and so that reinforces, again, that team-based care*” (FG-PD). Yet, while residents valued team members’ roles, they often expressed frustration when they could not address the issue themselves. As one resident described having to refer to a social worker, “*[i]t’s just like, oh, I don’t know how to take care of this, but I’ll find somebody who does*” which felt like a “*cop-out*” because “*I wish I at least could get the ball rolling and sort of assist in the process*” (FG-R1). Supervisors’ need for oversight played into this tension while residents looked for opportunities for autonomy in patient care. In their interaction with supervisors, the expectation that residents take ownership for ‘their own’ patients increased the tension between being a good team member and always being the team leader. Supervisors asked the resident “*to summarize the plan so they [the supervisors] are not like taking over the visit because it’s my [the resident’s] patient*” (FG-R2) to foster the professional growth for residents to more independently care for patients.

### Health system opportunities and limitations

Residents learned about system level impacts on patient care. One resident noted: “*part of the issue that makes it tough is that we, because of our structure of being here every six weeks pass it on*”, highlighting that schedules may prevent continuity and ownership of care, which may worry residents that things “*will fall through the cracks*” (FG-R2). This inter-visit disruption in continuity impacted resident perception of accountability as they “*feel responsible to like sign it out in an effective way which plus/minus happens*” since residents did not “*want to create work for other people so you try to do it yourself*” (FG-R2).

Our data suggest that the limitations of the health system may embolden residents to become advocates for their patients. For example, one faculty member recounted how a resident worked for three years to obtain an air conditioner for a patient with asthma, showing residents that “*[i]t’s not until you try something over and over again and then you actually figure out what the patient is capable of a month later*” (FG-F1). In assuming their role as advocates, residents learned firsthand about the challenges patients face in navigating the healthcare system. When one resident “*failed to make an appointment*” for his patient after making several calls, he noted frustration and helplessness: “*if I can’t do it, how are they going to do it? That makes me really angry, actually, because I realize that, like, yeah, it’s really difficult. They’re already holding on by a thread, and that just pushes them in the wrong direction*” (FG-R1).

Time constraints and caring for patients beyond the clinical encounter, for example, due to system scheduling processes along with the importance of longitudinal relationships between patient and resident was relevant to this domain and the patient needs domain. All three domains highlight the importance of residents learning the social determinants of health and how these affect the care of patients and possibly lead to or sustain healthcare disparities. Residents learned how the health system affects their patient directly and how to consider these factors when making a plan. For example, the amount of time allotted for seeing the patient may be dictated by the health systems billing processes or the patient has little or no insurance to cover needed medicines.

## Discussion

**Figure 1:**
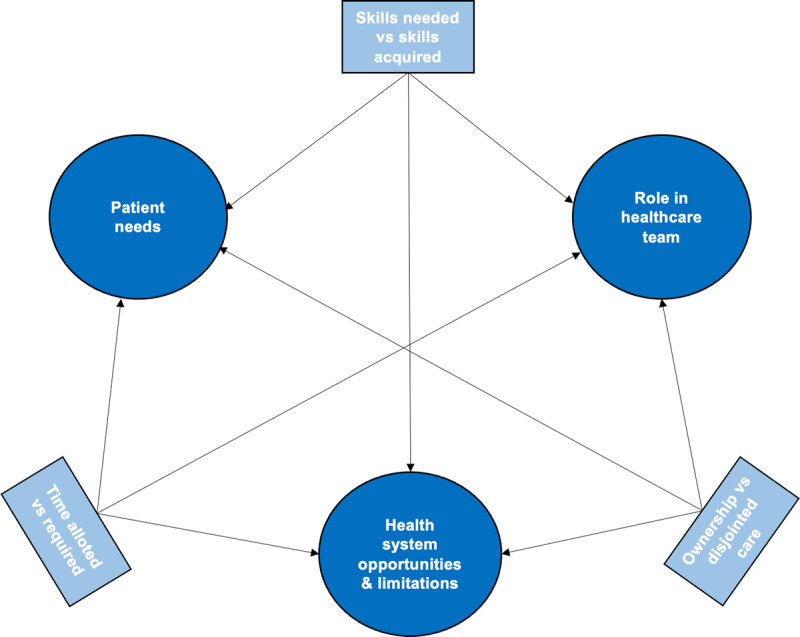
**Summary of content learned during ambulatory care rotations.** This schematic highlights the connection between the three main content domains of ambulatory learning (circles) and the tensions perceived within and between each of these domains (rectangles).

Through this case study, we found two main outcomes of what residents learn in the ambulatory setting. First, learning content for residents largely fell into three domains: patient needs, the resident’s role within a team, and healthcare system opportunities and limitations. Second, residents developed an understanding of their roles as physicians in ambulatory care via the tensions experienced within and between these domains. This was especially evident in how residents learned the significance of the health system and social determinants of health and their effect on the patient’s health. Figure 1 visually represents the domains and tensions that jointly constitute what residents learn in ambulatory care.

What was obvious in our case study was that what residents learned in the formal curriculum did not appear to adequately prepare them for practical aspects of patient care in the ambulatory care environment. For example, learning to care for medically and socially complex patients largely occurred informally via the hidden curricula in the ambulatory setting. To this end, it would be helpful to develop detailed ambulatory care competencies in the care of complex patients that involves the social determinants of health and relationship and trust building within teams as was previously demonstrated in the inpatient setting [17,19].

Additionally, we found residents learn that care delivery in the ambulatory setting is complicated by learning to be a member of a team, demonstrating the importance of the learning environment as well as the social nature of learning from a socio-cultural perspective [16,30]. While the residents in our study looked for ownership of patient care, that care is also shared with other team members and their desire to do the same within their professional roles [30,31]. This may be perceived as a lack of continuity on the part of residents, which may affect their sense of satisfaction with patient care. In general, the domain “role within the healthcare team” highlights that social interactions and context played a critical role in residents’ learning.

We found that opportunities and limitations of the health system have practical implications for resident learning. Seeing the importance of team-based care is contradicted by the inconsistent provider continuity while on other rotations, as described by Bates [32]. Residents in our study recognized that continuity in patient care has many benefits and yet acknowledged how the existing schedule structure during training limits accessibility and ownership, highlighting the tension between striving for ideal patient care while accepting the reality of practice. Historically, there has been more time spent in inpatient settings, leading to an appearance that the outpatient realm was not as important. Future research may involve investigating alternative scheduling strategies to mitigate the perceived lack of continuity in a system of working in blocks of inpatient and outpatient rotations.

There are several limitations in this study. Naturally, the context of this case study may differ from other ambulatory settings and residency programs. Nevertheless, the described characteristics of the research setting (e.g., large urban teaching hospitals with dedicated outpatient care facilities) may be present in many other contexts, and we invite readers to transfer applicable findings and insights from this study to their own setting. It is important for readers to evaluate their own local healthcare system and its effect on the learners. Nonetheless, we do expect that recognizing the tensions residents have to navigate would be helpful in any ambulatory learning environment. We suggest that the tensions we described could be a helpful starting point when transferring our findings to other contexts.

A second limitation might be the consequences of choices made while developing the moderator guide and omitting certain topics to foster the discussion. Despite these limitations, we are confident our results demonstrate *what* residents learned. This study demonstrates what the residents learned at this moment in time, and it may be interesting, and a point for further research, to look at their learning longitudinally as well as how they learn. Focusing on the socio-cultural framework in ambulatory care adds to what is known in the inpatient setting.

These findings suggest the importance of strengthening inter-professional education in ambulatory care to improve health outcomes for patients. Recognizing how informal and hidden curricula play into inter-professional learning and the social determinants of health are paramount to strengthen residents’ learning. For instance, ignoring barriers to care in the moment, such as food insecurity, will hamper residents’ learning and indicate that this is not an important area of concern for patient care. As seen from this work, relationships and point-of-care learning with their supervisors is critical to residents’ learning, so faculty development regarding socially complex patients is critical [33,34]. These findings suggest that either the system needs to be aligned with the experience of training or we, as medical educators, need to align our learning environments to the system. Such modifications could include restructuring resident schedules to account for socially complex patients and improve continuity, developing new competencies with a focus on complex patient care, aligning classroom-based didactic content with the realities of practice, and fostering inter-professional team-building in ambulatory care.

## Conclusions

This work revealed that in ambulatory care there were two outcomes of residents’ learning. First, resident learning encompassed three domains: 1) patient needs, 2) residents’ role within the healthcare team and 3) healthcare system opportunities and limitations. Second, residents develop an understanding of their role via three tensions among and between these domains. Residency training is a mix of inpatient and outpatient learning and learning in ambulatory care is an important aspect of resident learning and relatively understudied. Understanding this learning better enables more deliberate integration of the learning opportunities in ambulatory care and will complement the inpatient setting. Because ambulatory care is expanding continuously and, at present, much of what is learned by residents in ambulatory care is not included in the formal curriculum, it will be imperative to better align our curricula and learning environments to the learning needs of our residents.
